# Challenges in Clinical Research in Low and Middle Income Countries: Early Career Cardiologists’ Perspective

**DOI:** 10.5334/gh.1293

**Published:** 2024-01-25

**Authors:** Zainab Atiyah Dakhil, F. Aaysha Cader, Amitava Banerjee

**Affiliations:** 1 Ibn Al-Bitar Cardiac Centre, University of Baghdad, Baghdad, Iraq; 2 Kettering General Hospital, Kettering, United Kingdom; 3 Institute of Health Informatics, University College London, UK

**Keywords:** Developing countries, Research career, Mentorship, Sponsorship

## Abstract

Despite a growing burden of cardiovascular diseases (CVDs) in Low and Middle Income Countries (LMICs), there remain significant barriers to researchers living in these countries regarding the initiation, progression and completion of research. These obstacles are multifactorial, ranging from a lack of general incentives, national and institutional initiatives and capacity, limited opportunities for funding, and lack of mentorship and support for the presentation and publication of research. In this perspective piece, we highlight some of the challenges we have observed from our experience as early career cardiologists in LMICs and present some potential solutions to address these issues.

## Introduction

Despite a greater burden of cardiovascular diseases (CVD) and non-communicable diseases (NCD) in Low and Middle Income Countries (LMICs), there remains a profound lack of research output from these countries [1,2]. Locally-led and locally-implemented research are of paramount importance not only from mechanistic standpoints, but also to identify implementation methods unique to sociodemographic settings in LMICs. This issue was especially eye-opening at the World Heart Federation Emerging Leaders Programme, which brought together multiple young researchers from diverse backgrounds. There are many obstacles and challenges in both building a research infrastructure, as well as the progression of cardiovascular research in LMICs. Barriers include a lack of established research infrastructure and mentors, inadequate training, capacity-building, funding opportunities, language barriers to the publication and presentation of research outputs, and travel restrictions to attending conferences [3]. As early career cardiologists in LMICs keen on fostering a research culture in the cardiovascular landscape, we highlight here the need for locally-led research and some of the challenges we have experienced and offer some potential solutions to address these issues.

Better data and better research lead to better patient outcomes. In order for LMICs to action this and to have capacity to do this, young researchers (especially doctors) are crucial to leadership and implementation (which leads to more opportunities for researchers in LMICs to own and lead their research agendas).

## The Need for Locally Led Research in LMICs

The burden of non-communicable diseases, and especially cardiovascular diseases continues to rise in LMICs [4]. Differences in the pathophysiology and progression of disease, and the metabolism of certain drugs specific to ethnicities exist [5,6]; there is therefore a need for more data from LMICs wherein these ethnicities are predominant. It is undisputed that better data lead to better patient outcomes. As such, locally-led cardiovascular research in LMICs is crucial, not only in identifying specific disease patterns in CVD (e.g. increased burden of young acute coronary syndromes in South Asia), but also to inform effective implementation strategies within LMICs [7]. In doing so, the involvement of young researchers (especially doctors) is crucial to leadership and implementation, however there are many challenges encountered.

## Research Landscape, Training, and Capacity-building

Central to the problem is the fact that, in many LMICs, the concept of a clinician-scientist or clinician-researcher, including in cardiovascular diseases, is unfamiliar. Given the large patient to doctor ratios, physicians are often extremely busy with their clinical practices, with many undertaking private practice after regular hours as well. Research and clinical medicine are often siloed, and often, LMICs physicians are only truly introduced to concepts, conduct and practical aspects of good research during their postgraduate education. This rather late step too, is often undertaken as a mandatory requirement towards fulfilling their degrees and academic promotions, and rarely followed up with further research on the topic, partially owing to there being little incentive to do so, as it is often under-appreciated and undervalued.

Thus, there are limited original data on prevalence, management practices and outcomes of CVD, specific to many LMICs populations, with even fewer randomised controlled trials [8]. As a result, there is little local data to base the formulation of local clinical practice guidelines, which are thus often derived from those written in established high-income countries (HIC).

This problem requires intervention at national, institutional and professional society level, with the involvement of multiple stakeholders to appreciate and prioritise the research in these countries, specific to these populations, thus spurring on the conduct of meaningful regional research and capacity-building for the future.

## Established Research Culture & Infrastructure

The lack of an established research infrastructure and poor integration of a research culture within organisations, institutionally and nationally, present a major obstacle to early career physicians interested in progressing with research projects. Often there are no established teams within institutes, including a lack of research assistants, statisticians and appropriate information technology and software systems for analysis. In many LMICs healthcare settings, electronic health records (EHR) are not in routine clinical practice [9], thus precluding any form of well-established databases that can be used to extract variables for research, which often constitute a starting point for data analysis and research opportunities especially for aspiring researchers. Often, even retrospective data collection is acquired manually, by physician-investigators themselves, given that only few centres, even among universities and research institutes, employ dedicated research assistants to undertake this job.

The volume of patients in LMICs offers immense potential to explore solutions to problems endemic to our own countries. Thus, building research capacity is essential by encouraging local institutes to set up their own research units, starting small, and gradually developing a network of local and international collaborators. Digitalisation of data, even the building of databases on platforms such as *Red Cap* will undoubtedly provide potential for observational research of relevant questions in large sample sizes.

## Lack of Protected Research Time

A lack of protected research time means that much of the research work is undertaken after regular work hours. One might argue that some of the tasks might be delegated to medical students or potential junior colleagues, however, this manpower is also limited, as research efforts are rarely rewarded proportionately, with the predominant focus being clinical skills; thus, there is less incentive for those at the inception of their careers to get involved, unless they exclusively proceed to research careers as higher degrees. Given this difficulty in task delegation, researchers have significantly less output and productivity, which can be especially challenging when navigating particularly early career challenges, often delaying publications, and increasing abstract presentation to publication time.

The formation of collaborations with like-minded individuals can be invaluable in reducing individual workloads. Establishing professional social media profiles on platforms such as *ResearchGate* and *LinkedIn*, and using them appropriately to reach out to senior colleagues and identify juniors interested in working as research assistants, can be especially useful in simplifying the tasks of delegation and collaborative work.

## Funding Opportunities

Central to the issue of protected research time, is a lack of funding. While HIC early career researchers may benefit from multiple grant opportunities from governments, national cardiovascular societies, and charities, this is often not the case in LMICs [10]. Additionally, there remain geographic disparities in global grant funding, thus further depriving LMICs researchers [11,12]. Many of the funding opportunities in LMICs, and indeed those derived from international bodies are directed towards large-scale public health, community interventions and infectious diseases research. Furthermore, industry funding, especially those from interventional cardiology device companies are often restricted to HIC, as many LMICs do not have the trials infrastructure, state-of-the-art cath lab facilities and expertise to implant novel devices nor conduct phase 2 drug trials.

A democratisation of funding is necessary, with professional societies investing exclusively in LMICs research and building capacity. LMICs researchers can also look out for funding opportunities that involve collaborations with HIC.

## Collaboration and Mentorship

In general, while collaborative projects with researchers from HIC exist, these are often limited to large-scale public health and community interventions. Given the high burden of communicable diseases too, these projects often take precedence, however, the rising burden of CVD, and particularly young Acute Coronary Syndromes in many LMICs should not be ignored. Furthermore, research in what are often perceived as ‘niche’ specialities like interventional cardiology are rarely funded in LMICs, owing to far more pressing health issues. It is an oft-discussed fact that LMICs researchers are hired for predominantly time-bound projects of interest by collaborators from the ‘Global North’, thus resulting in a significant lack of capacity-building for the future. Issues such as authorship, where LMICs-specific research continues to be led and published with HIC researchers as first and senior author, are testament to this [13]. In addition, the lack of capacity and research infrastructure is often what precludes LMICs from recruiting into major cardiovascular trials, representing missed opportunities for early career cardiologists interested in such research to gain training as site principal investigators.

Even as postgraduate students, there is a scarcity of good local mentorship for aspiring researchers, mainly owing to a lack of established research structure and senior colleagues well-trained in research methods. Mentorship and sponsorship are crucial to developing good research careers, particularly alongside busy clinical careers. Thus, many LMICs researchers are not afforded the opportunities derived through their mentors’ networks. One potential solution is to reach out to overseas mentors directly, via social media and other connections, and establish collaborations and opportunities for mentorships.

## Publication and Presentation of Research Outputs, Including Language Barriers

Further to this general lack of training in research methodology and mentorship, many LMICs researchers face the additional challenge of language barriers, as many are not native speakers of English, the predominant form of scientific communications. They are therefore limited in terms of effective communication of their research in the form of scientific publications, manuscripts and oral presentations. A lack of funding precludes submission to some high impact factor journals, which often may not waive article processing fees. The language issue is further compounded by additional fees incurred for paying professional language editors, as there may not be resources or people locally who are able to do this free of cost.

Potential solutions include dedicated training of researchers to build these skills, by academic organisations and universities. Additionally, journal editorial boards could potentially provide professional language services for free or at minimal cost, while also considering waivers of APC, as important research by LMICs may be overlooked owing to these issues. Journals can also offer internships and reviewer training programmes, where researchers gain an insight into the peer review system, as well as learn from a paired mentor, who will provide critical feedback for continued learning.

## Travel for Conference Attendance and Speaking Engagements

Many LMICs researchers require visas to travel to a vast majority of countries. Thus, even when their research is accepted as abstracts for presentation, the obstacles are massive. Often, visas are delayed, or not granted, precluding travel and thus lost opportunities to speak, engage, and critically network and find collaborators and potential mentors. The need for visas requires considerable planning, allocation of time and resources, and precludes LMICs researchers from attending multiple meetings a year, owing to time lag that mandatorily needs to be allowed for visa procurement. We have often had to pass up opportunities for attending meetings owing to these issues, even where funding was granted. Travel and visa costs can often be expensive, especially given the uncertainty of travel dates, on top of a lack of funding and LMICs clinician pay scales, which are additional barriers. Partially as a result of this, researchers from LMICs are significantly under-represented in high impact cardiology meetings and journal publications [14,15]. Travel and educational grants by global societies and cardiovascular organisations can help in overcoming some of these challenges to enable investigators from LMICs to showcase their work [15]. Professional societies must also consider hybrid components to meetings and conferences, thus that those unable to travel in-person are not precluded opportunities to serve as faculty and speakers.

Global organisations play a key role in advancing the careers of LMICs researchers by offering training programs, networking opportunities and travel grants. The World Heart Federation Salim Yusuf Emerging Leaders Programme, is an excellent example: the programme brings together young researchers from diverse backgrounds across the world in each year’s cohort, and provides unique opportunities for learning, mentorship, action-oriented research training in implementation science by global leaders, networking, building and executing a project with seed funding, leading up to publication and meaningful global impact [16]. Noting the key advantages of in-person networking, the programme also offers travel grants for participants from LMICs without institutional travel support to join in-person, including support with visa applications. Indeed, this paper, and much of its content were precisely issues that were discussed by the authors on the side-lines of the think tank meeting in Lisbon, in 2021.

## Conclusion

**Figure 1:**
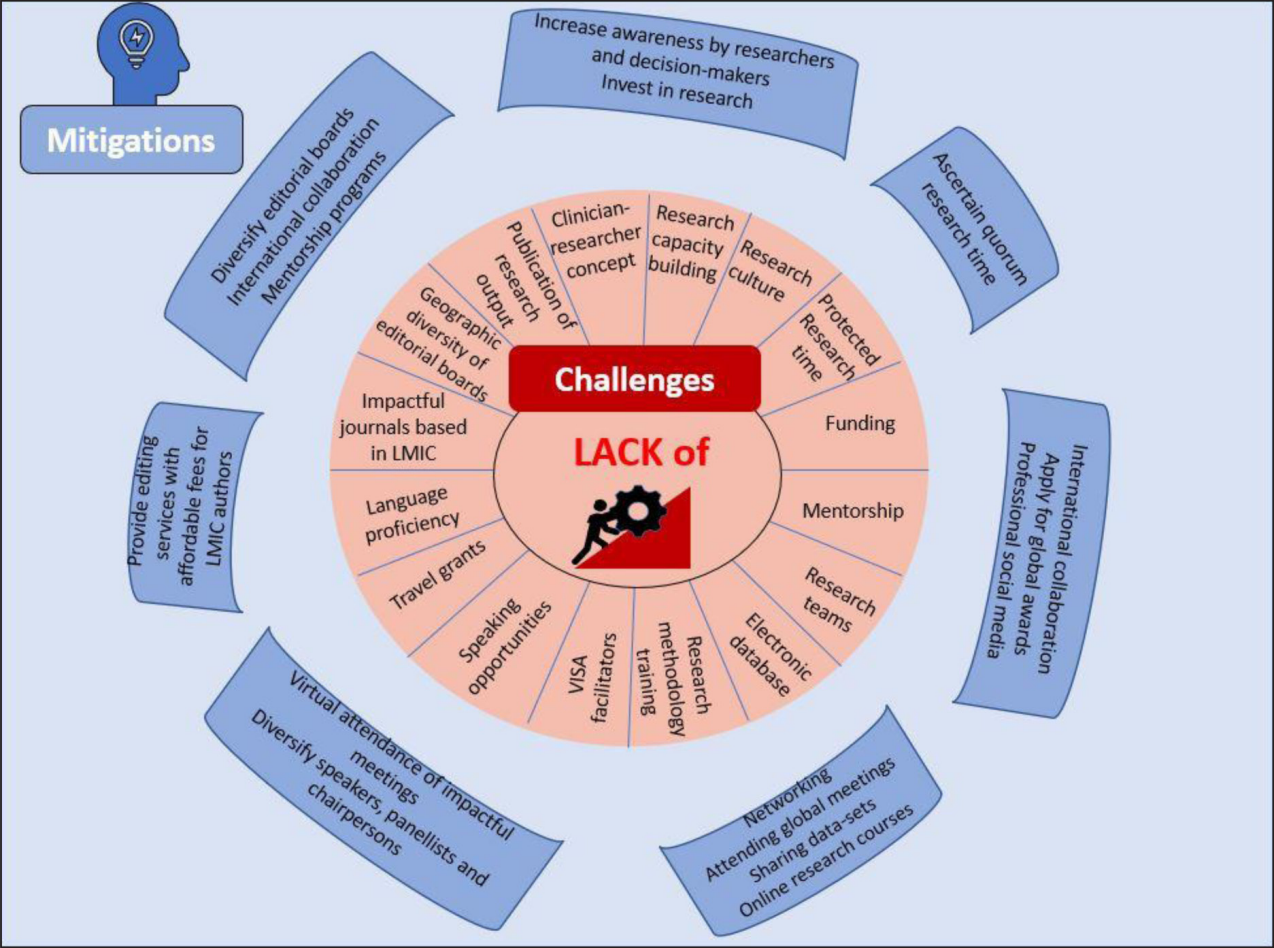
Challenges and mitigations for research in low-and-middle-income countries (LMICs).

While challenges to research in LMICs exist, there are certainly many solutions that can be offered, Figure 1. During the COVID-19 pandemic, we learnt to pivot, and saw a flattening of economic hierarchies, with researchers across the globe receiving opportunities to network, collaborate and learn across virtual platforms. While the challenges might seem insurmountable, accepting the need for change, and pivoting in that direction are essential. The key for initiating and maintaining research ecosystem in LMICs is by streamlining research with the usual medical care as a way for sustainable long-term change.
